# Surface Roughness after Milling of the Al/CFRP Stacks with a Diamond Tool

**DOI:** 10.3390/ma14226835

**Published:** 2021-11-12

**Authors:** Elżbieta Doluk, Anna Rudawska, Józef Kuczmaszewski, Izabela Miturska-Barańska

**Affiliations:** Department of Mechanical Engineering, Lublin University of Technology, 20-388 Lublin, Poland; a.rudawska@pollub.pl (A.R.); j.kuczmaszewski@pollub.pl (J.K.); i.miturska@pollub.pl (I.M.-B.)

**Keywords:** sandwich construction, milling, surface roughness, surface topography, SEM analysis

## Abstract

This study presents the results of research on the surface quality of hybrid sandwich structures after milling with a diamond blade tool. It identifies the effects of feed and machining strategy on the roughness and topography of the surface. It provides an analysis of Ra and Rz surface roughness parameters as well as Sp, Sz, and Sv surface topography parameters. The processed object was a two-layer sandwich structure consisting of aluminium alloy 2024 and CFRP (carbon fibre-reinforced polymer) composite. The minimum values of the Ra and Rz surface roughness parameters were obtained on the aluminium alloy surface, whereas the maximum values were obtained on the CFRP surface. The same was true for the 3D surface roughness parameters—the lowest values of Sp, Sz, and Sv parameters were obtained on the surface of the metal layer, while the highest values were obtained on the surface of the composite layer (the maximum value of the Sp parameter was an exception). A surface topography analysis has revealed a targeted and periodic pattern of micro-irregularities for the vast majority of the samples considered. The statistical analysis shows that the surface roughness of the aluminium alloy was only affected by the feed rate. For the CFRP, the feed rate and the interaction of milling strategy and feed rate (S × f_z_) had a statistically significant effect. The obtained results provide a basis for designing such sandwich element processing technology, for which differences in roughness and topography parameters for the component materials are lowest.

## 1. Introduction

Sandwich structures are an example of a group of engineering materials that are growing in popularity. Their relatively low weight makes them increasingly popular in many areas of technology [1]. Sandwich structures are made up of two basic components: a thinner outer layer (face) made of a more rigid material with more favourable strength properties and a thicker and lighter inner layer (core) made of a different material. The layers combined in this way make up a lightweight, rigid, and robust structure with more desirable strength properties compared to equivalent solid structures. The idea behind layered material is that the faces are made of high-strength materials and the core is made of a less rigid material. This is due to the fact that the faces usually carry bending and compressive loads, including impact loads; thus, high rigidity and strength is required. The primary purpose of the core is to carry shear and compression loads, which means that it does not need to have the same strength properties as the shell. The core has a relatively low density, which ensures high bending strength and high stiffness compared to the overall density of the panel—sandwich panels are typically used as structures that carry bending loads. Layered composites ensure a reduction in lateral deformation of the structure and increase its resistance to buckling [2].

The machining of hybrid sandwich composites, compared to the machining of metal objects, is more difficult due to the heterogeneity and anisotropy of the structure resulting from the combination of materials with different mechanical and physical properties. The more varied the layer materials, the more difficult the processing. Changing the properties of the materials that make up the structure, in a short interval of time, leads to a non-uniform performance of the sandwich composite. Therefore, it is a major challenge to properly select the tools and technological parameters that would enable the machining of this type of materials [3,4].

Polycrystalline Diamond (PCD) tools are often used in the machining of hybrid sandwich structures. These tools are characterised by high strength and sharp edges for precision machining. In addition, diamond coatings are used due to their good thermal conductivity, chemical inertness to resins at elevated temperatures, and low friction coefficient [5].

Surface roughness is one of the more frequently analysed indexes in machining. This is because it affects the functional properties of the processed item, adhesion of coatings, wear, ability to distribute lubricants, or heat conductivity [6]. Sufficiently low surface roughness is often an important factor in the way in which a structure is used. For hybrid sandwich structures, homogeneous surface quality after treatment is a very important issue. This is due to the fact that structures used in the aerospace and automotive industries are frequently covered with technical, protective, or decorative coatings. Varying the surface roughness of the materials forming the sandwich structure hinders adhesion of the coatings, compromises the visual effect, and may make it difficult to fit the components together, e.g., when the structure is joined to form large panels.

A literature analysis shows that most of the attention is focused on investigating the machinability of fibre composites (mainly CFRP) and the quality of holes made in hybrid sandwich structures [7,8]. In [9], the effects of cutting speed, feed, and depth of cut and cutting tool blade angle on the surface roughness and formation of burrs during milling of Ti/CFRP structures were investigated. It was demonstrated that the CFRP layer had a higher surface roughness, while the Ti layer had a higher proportion of burrs. The authors of [10] present the technique for the self-adjustment of cutting parameters when drilling holes in sandwich composites. Palanikumar [11] studied the effect of cutting parameters (cutting speed, feed rate, and cutting depth) on surface roughness after GFRP (glass fibre-reinforced polymer) processing with a PCD tool. Based on the results, he developed a model to predict surface quality after machining GFRP composites. The authors of [12] also investigated the influence of technological parameters (rotational speed, feed per blade) and tool type on the cutting strength and surface roughness after milling CFRP. Based on the analyses, it was found that feed rate had the greatest effect on the dependent variables studied. The uncoated cutter produced the lowest surface roughness values and lower cutting strength. Surface roughness increased with the increasing number of chip grooves of the tool and the inclination angle of the cutting edge. The authors of [13] investigate the effects of cutting speed, feed rate, and the presence of a tool coating (TiAlN) on surface quality and cutting strength after milling an Al/CFRP structure with a carbide tool. Mueller-Hummel et al. [14] compared the machinability of a CFRP and an aluminium alloy. They developed simplified machining models to avoid or reduce the occurrence of common defects in the composite material. They also presented the influence of cutting tool geometry (cutters and drills) on the quality of machined surfaces.

Considering that most works deal with the surface quality of only fibre composites or the hole quality and wear mechanisms after drilling of sandwich composites, it seems reasonable to try to determine the influence of machining parameters and strategies on the surface quality of hybrid sandwich structures after the milling process. The present study evaluates the surface roughness and topography of Al/CFRP structures after machining with a tool with polycrystalline diamond blades.

## 2. Materials and Methods

**Figure 1:**
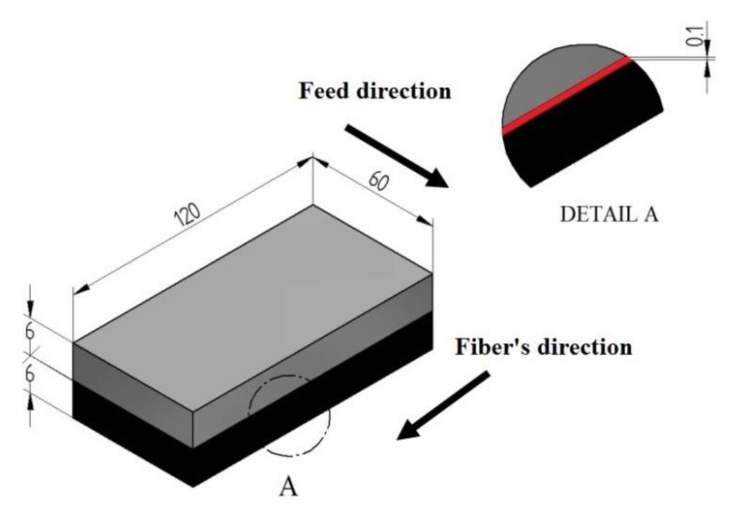
Sample shape and dimensions.

The study examined a 2-layer sandwich structure made of aluminium alloy 2024 (Al) [15] and a carbon fibre-reinforced polymer (CFRP). These materials were chosen due to their frequent use in the aerospace industry. The shape and dimensions of the sample are shown in Figure 1.

CFRP consisted of unidirectional CM-Preg TI02 20/1000 CP00690 (trade name) prepregs by C-M-P company (Heinsberg, Germany) made of a thermoplastic epoxy matrix and reinforcement (60%) in the form of high-strength carbon fibres. The composite used in the study was formed by pressure-vacuum impregnation using a Scholz autoclave (Coesfeld, Germany). The material was cured at 130 °C under 0.4 MPa pressure for 1 h.

The tested sandwich structure was created by bonding the materials together using Scotch-Weld-9323 B/A structural adhesive (3M, St. Paul, MN, USA) mixed at a ratio of 100:27. The polymerisation process proceeded in a vacuum bag under 0.1 MPa pressure at room temperature for a period of 24 h. After that time, the samples were seasoned for 7 days in ambient conditions. The polymerisation and seasoning processes were carried out at 23 ± 1 °C and 35% ± 2% humidity. The thickness of the adhesive was 0.1 ± 0.02 mm (Figure 1).

**Figure 2:**
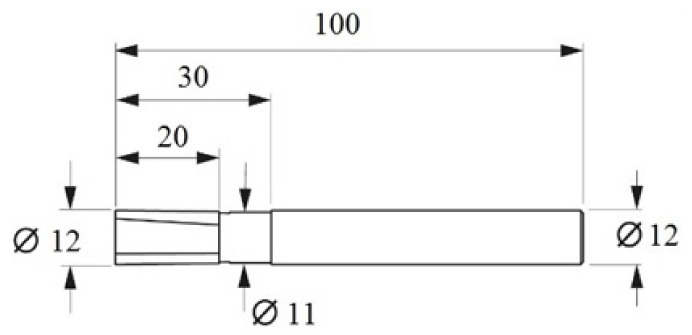
Twin blade shank cutter with PKD cutting edge [16].

The study involved a circumferential concurrent milling process of the shorter edge of the specimen (60 mm—Figure 1). The machining was performed on a VMC 800 HS vertical machining centre (AVIA, Warsaw, Poland). The machining was carried out using a double-edged shank cutter with a diamond blade with straight toothing (α = 45°, λ_s_ = 0° and γ_s_ = 0°) and a workpiece diameter of Ø12 mm (Hoffman Group, Munich, Germany). The dimensions of the tool are shown in Figure 2. The cutting edge material has been selected due to its very good properties. Diamond tools are characterised by high blade durability and, due to their sharp cutting edges, they provide high surface quality after machining. Tool geometry also affects surface quality. Straight cut tools are recommended for processing sandwich materials. This cutter geometry theoretically does not cause repulsion of the tool from the part being machined, which reduces the occurrence of surface defects, including delamination and tearing of the composite material. A higher value of the cutting edge inclination angle leads to burrs and chipping.

**Figure 3:**
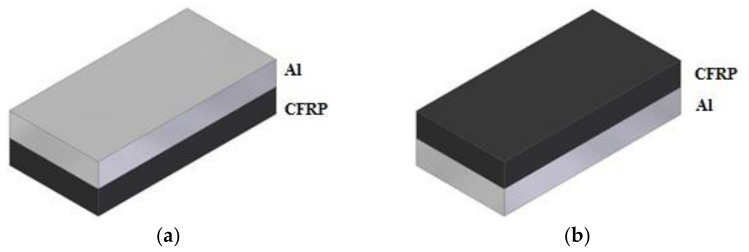
Considered milling strategies: (**a**) Al/CFRP, (**b**) CFRP/Al.

The influence of cutting parameters (f_z_ feed rate) and machining strategy (Al/CFRP and CFRP/Al) was studied in the experiment. The samples were milled with three variable feed rates: f_z_ = 0.04 mm/blade, f_z_ = 0.08 mm/blade, and f_z_ = 0.12 mm/blade. The range of the feed was selected to determine the effect of the increasing the feed on the surface roughness—a higher feed rate increases the machining efficiency and reduces the machining time, which is desirable. Two machining strategies were also applied during machining: (i) Al/CFRP strategy—milling was started from the metal layer; and (ii) CFRP/Al strategy—milling was started from the composite layer (Figure 3).

**Figure 4:**
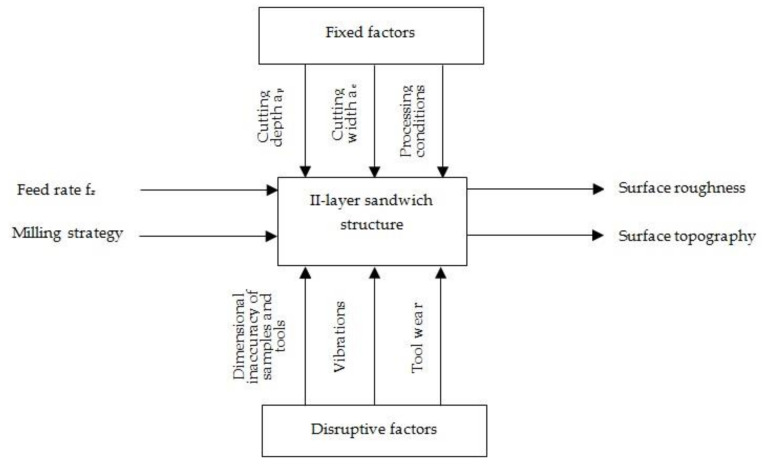
Experiment plan.

The machining was performed with a constant cutting depth a_p_ = 12 mm and a constant cutting width a_e_ = 4 mm. The value of a_p_ parameter was chosen to allow for the simultaneous cutting of both layers of the structure. Each sample was processed three times—the analysed results were the arithmetic mean of the measurements of the three samples. Milling was performed without the use of machining fluid. Figure 4 shows the experiment plan. Fibre composites fall into the category of hard-to-machine materials [17]. The most frequent forms of damage to this type of material include delamination, fibre pulling, and warp cracking. It has been shown in [18,19] that the most suitable surface roughness parameters for fibre composites, considering the aforementioned forms of damage, are Rz (maximum height of surface roughness profile) and Rt (total height of surface roughness profile). Bearing the above in mind, the parameter Rz [20] and due to the metal layer the parameter Ra (arithmetical mean height) [20] were recorded in this study. For the surface topography, measurements were made for the following parameters: Sz (ten-point surface roughness height), Sv (maximum surface indentation height), Sp (maximum surface elevation height), Sku (kurthosis), and Sds (summit density) [21].

SEM analysis was applied to evaluate the surface of the samples after milling. A Phenom ProX scanning microscope (Thermo Fischer Scientific, Waltham, MA, United States) (5–15 kV, high vacuum) was used for this purpose.

## 3. Results

**Figure 5:**
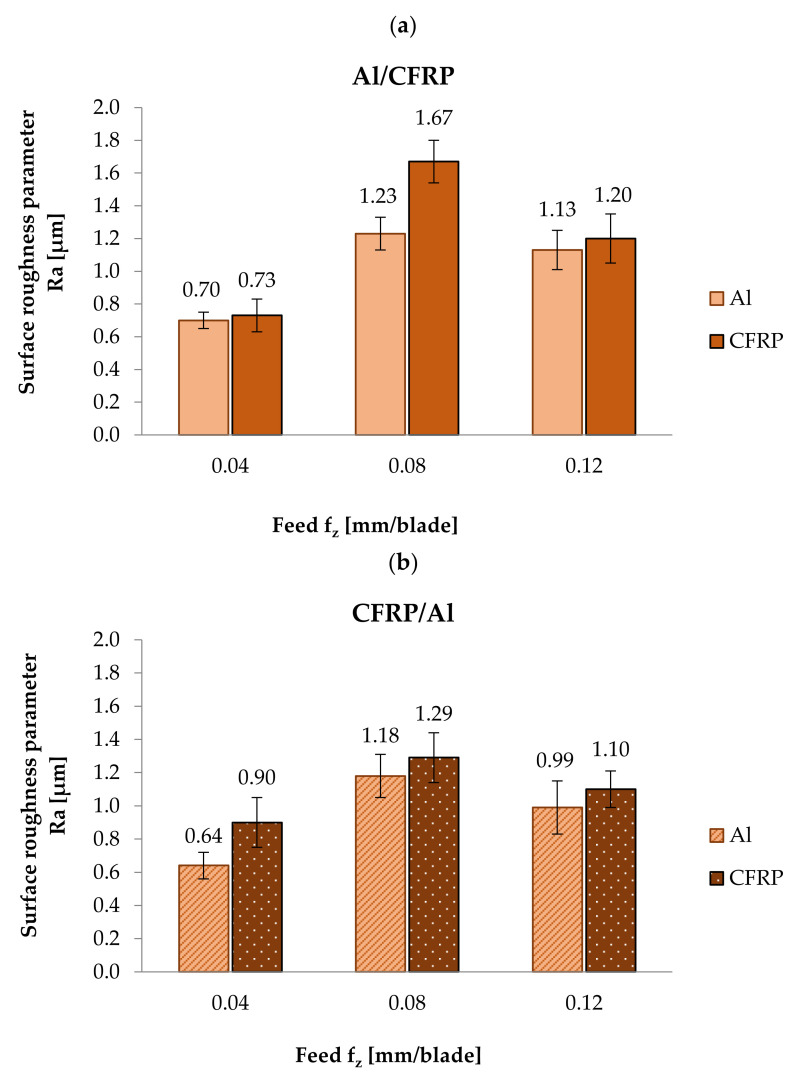
Ra parameter measurement results after milling: (**a**) using Al/CFRP strategy, (**b**) using CFRP/Al strategy.

Figure 5 presents the results of the Ra parameter measurements obtained on the surface of the aluminium alloy and CFRP after milling using Al/CFRP (Figure 5a) and CFRP/Al (Figure 5b) machining strategies.

The minimum value of the Ra surface roughness parameter obtained after treatment with the Al/CFRP machining strategy was observed on the surface of the metal layer (0.70 µm) for f_z_ = 0.04 mm/blade. The maximum value of the Ra parameter for this milling strategy (1.67 µm) occurred on the CFRP surface for f_z_ = 0.08 mm/blade. The difference between the highest and the lowest values of the Ra parameter was approximately 58% (Figure 5a). For the CFRP/Al strategy, the highest and lowest results were obtained for the same feed rate values as for the Al/CFRP strategy: the minimum Ra value was recorded on the aluminium alloy surface (0.64 µm) for f_z_ = 0.04 mm/blade, and the maximum was recorded on the CFRP surface (1.29 µm) for f_z_ = 0.08 mm/blade. The difference between the results was nearly 50% (Figure 5b). A similar trend was observed for both machining strategies: on the surfaces of aluminium alloy and CFRP, the minimum values of the Ra surface roughness parameter were obtained after machining with a feed rate of f_z_ = 0.04 mm/blade; then, at f_z_ = 0.08 mm/blade, the values of Ra parameter increased (maximum values), and at f_z_ = 0.12 mm/blade, they decreased.

Analysis of the effect of milling strategy shows that for a feed rate of f_z_ = 0.04 mm/blade, the CFRP/Al strategy produced a decrease in the surface roughness of the aluminium alloy and an increase in the surface roughness of the CFRP. At feed rates of f_z_ = 0.08 mm/blade and f_z_ = 0.12 mm/blade, the CFRP/Al strategy resulted in lower Ra values for both materials.

**Figure 6:**
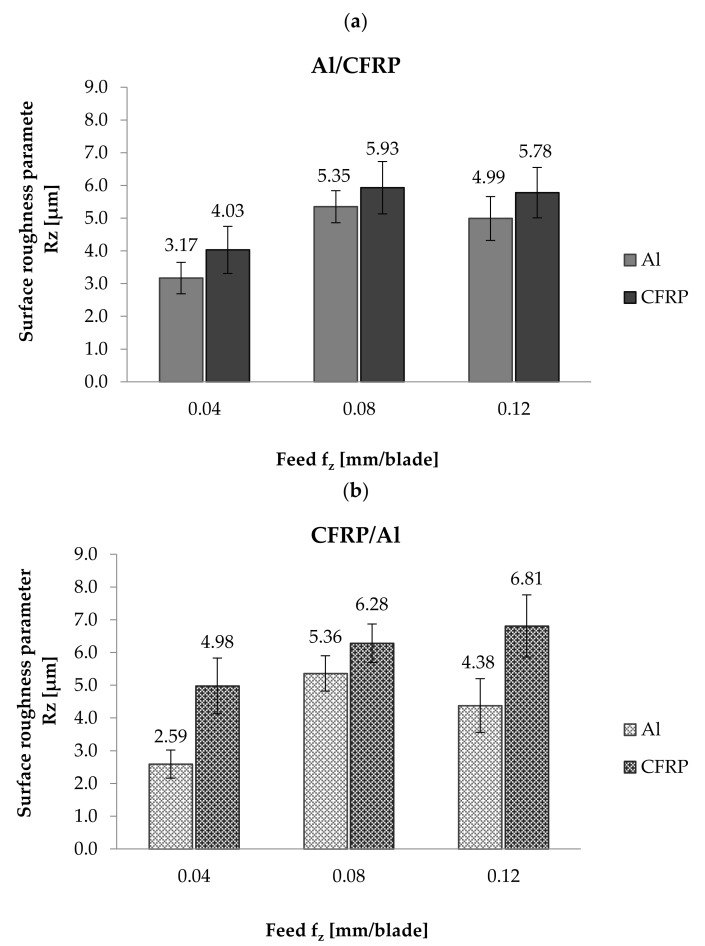
Rz parameter measurement results after milling: (**a**) using Al/CFRP strategy, (**b**) using CFRP/Al strategy.

The minimum value of Rz surface roughness parameter (3.17 µm) after treatment with the Al/CFRP strategy was obtained on the aluminium alloy surface for f_z_ = 0.04 mm/blade, and the maximum value (5.93 µm) was obtained on the CFRP surface for f_z_ = 0.08 mm/edge (Figure 6a). The difference between the highest and the lowest values of the Rz parameter was around 47%. The measurement results for the Rz parameter for the Al/CFRP strategy were similar to the results obtained for the Ra parameter: for both materials forming the sandwich, the lowest values of the Rz parameter were observed after milling with the lowest value of feed rate, then for a feed rate of f_z_ = 0.08 mm/blade, the Rz parameter reached its maximum values, which decreased at f_z_ = 0.12 mm/blade.

When using the CFRP/Al machining strategy, the minimum value of the Rz parameter (2.59 µm) was obtained on the surface of the metal layer milled at a feed rate of f_z_ = 0.04 mm/blade, and the maximum value (6.81 µm) was obtained on the surface of CFRP using a feed rate of f_z_ = 0.12 mm/blade. The difference between the values was around 62%. The values of the Rz parameter for the aluminium alloy increased and decreased alternately with the change in the feed rate. For the CFRP, an increase in feed rate resulted in an increase in the value of the Rz parameter (Figure 6b).

The CFRP/Al milling strategy for f_z_ = 0.04 mm/blade and f_z_ = 0.12 mm/blade, compared to the results obtained after milling using the Al/CFRP strategy, resulted in a decrease in the value of the Rz parameter measured on the aluminium alloy surface and an increase in the value of this parameter for the composite layer. At a feed rate of f_z_ = 0.08 mm/blade for both materials, the CFRP/Al strategy led to increased surface roughness.

Two-factor ANOVA was performed for statistical verification of the obtained results. The dependent variables were the Ra and Rz parameter values, whereas the milling strategy (S) and feed rate (f_z_) were the grouping factors. The analysis was performed with a confidence level of α = 0.05. Table 1 and Table 2 summarise the results of the conducted ANOVA variance analysis.

The data in Table 1 indicate that for the parameter Ra measured on the surface of the aluminium alloy, a highly statistically significant result was obtained only for the feed rate (F_5, 12_ = 10.26; *p* < 0.01). The milling strategy and S × f_z_ interaction reached similar values of the F test statistic and, from the perspective of statistical analysis, had no significant effect on Ra parameter. For the Ra parameter obtained on the surface of the CFRP, highly statistically significant results were obtained for the feed rate (F_5, 12_ = 27.95; *p* < 0.01) and S × f_z_ interaction (F_5, 12_ = 4.98; *p* = 0.04), where the feed rate had the greatest influence on the analysed Ra.

Table 2 lists the results of ANOVA variance analysis obtained for the Rz surface roughness parameter. For the aluminium alloy, the feed rate was the only factor that affected the values of the Rz parameter (F_5, 12_ = 12.70; *p* < 0.01). Highly statistically significant results for the CFRP were obtained for feed rate (F_5, 12_ = 27.22; *p* < 0.01) and S × f_z_ interaction (F_5, 12_ = 4.48; *p* = 0.04). For this material, the feed rate was the factor that most strongly influenced the Rz surface roughness parameter.

**Figure 7:**
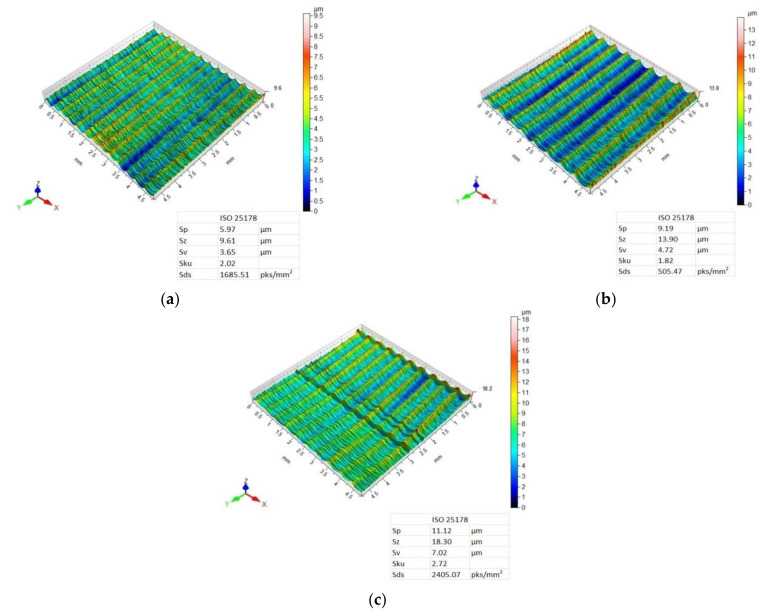
Surface topography of aluminium alloy after milling using Al/CFRP strategy and feed rate: (**a**) f_z_ = 0.04 mm/blade, (**b**) f_z_ = 0.08 mm/blade, (**c**) f_z_ = 0.12 mm/blade.

**Figure 8:**
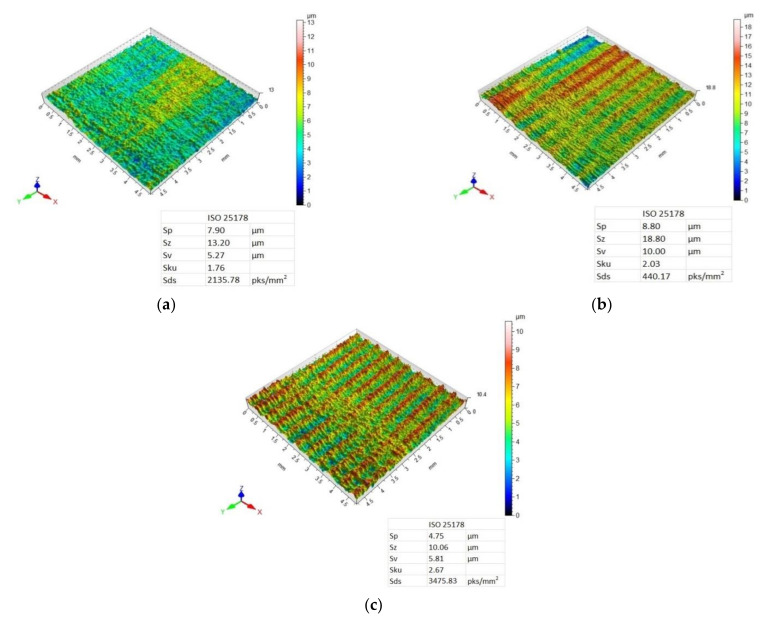
Surface topography of CFRP after milling using Al/CFRP strategy and feed rate: (**a**) f_z_ = 0.04 mm/blade, (**b**) f_z_ = 0.08 mm/blade, (**c**) f_z_ = 0.12 mm/blade.

**Figure 9:**
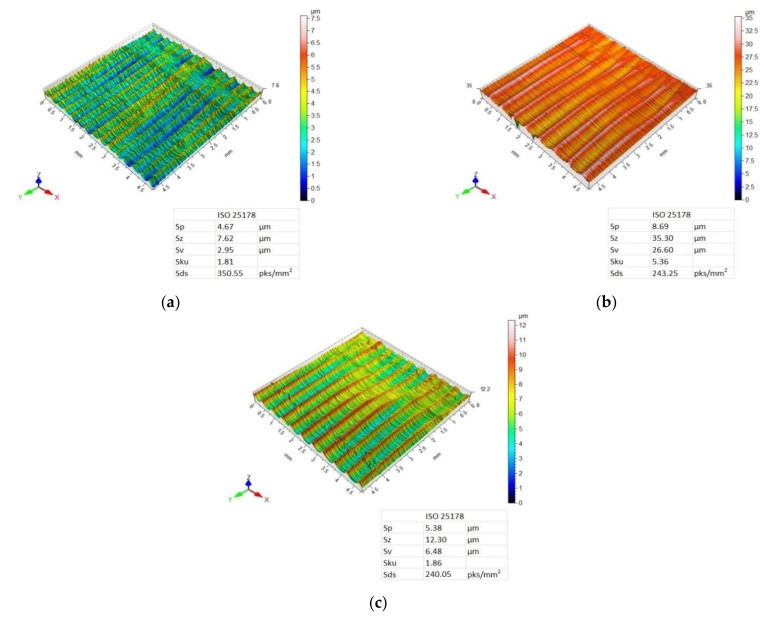
Surface topography of aluminium alloy after milling using CFRP/Al strategy and feed rate: (**a**) f_z_ = 0.04 mm/blade, (**b**) f_z_ = 0.08 mm/blade, (**c**) f_z_ = 0.12 mm/blade.

**Figure 10:**
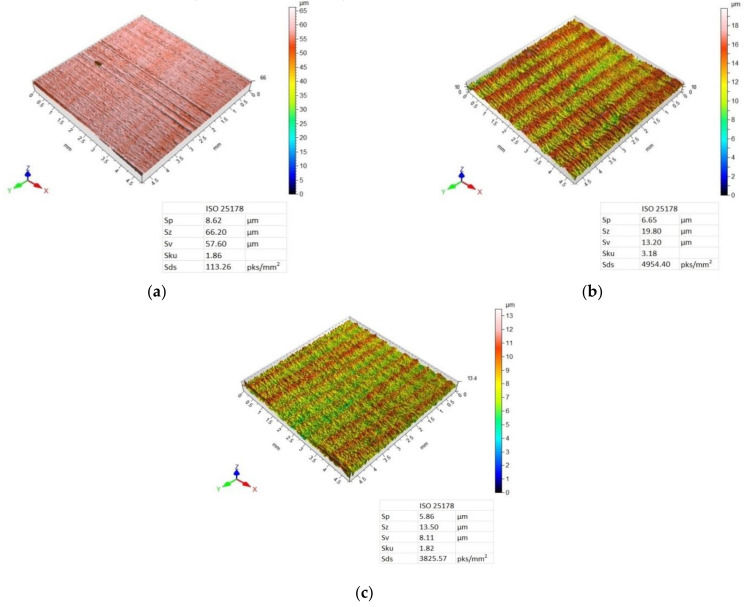
Surface topography of CFRP after milling using the CFRP/Al strategy and feed rate: (**a**) f_z_ = 0.04 mm/blade, (**b**) f_z_ = 0.08 mm/blade, (**c**) f_z_ = 0.12 mm/blade.

Figure 7, Figure 8, Figure 9 and Figure 10 present 3D maps showing the topography of the aluminium alloy and CFRP surfaces after milling. They present surface topography of each of the layers forming the considered sandwich structure, depending on the feed value and the applied milling strategy.

Three-dimensional (3D) images of the aluminium alloy surface after machining using the Al/CFRP strategy are characterised by a similar pattern of micro-irregularities—the structure of all surfaces is directional, and grooves and ridges that differentiate surface quality occur in a periodic manner (Figure 7). Values of the Sp, Sz, and Sv parameters increased with the increasing feed rate—the highest values of the considered 3D surface roughness parameters were recorded for the maximum feed rate (f_z_ = 0.12 mm/blade). For all feed values, Sku < 3, which means that the height distribution is skewed above the mean plane. The highest value of the Sds parameter was obtained after milling with the feed f_z_ = 12 mm/blade.

The surface topography of the CFRP after milling using the Al/CFRP strategy and feed rate f_z_ = 0.04 mm/blade is characterised by a disorderly arrangement of micro-irregularities (Figure 8a). For f_z_ = 0.08 mm/blade and f_z_ = 0.12 mm/blade, targeted and periodic traces of micro-irregularities are visible—grooves and bumps occur at similar intervals (Figure 8b,c). The minimum values of Sp and Sz parameters were obtained for a feed rate of f_z_ = 0.12 mm/blade, and the maximum values were obtained for a feed rate of f_z_ = 0.08 mm/blade. For the Sv parameter, the lower value was observed after milling with a feed rate of f_z_ = 0.04 mm/blade and the highest value was observed after machining with a feed rate of f_z_ = 0.08 mm/blade. Milling the Al/CFRP structure using a feed rate of f_z_ = 0.04 mm/blade resulted in lower Sp, Sz, and Sv values on the aluminium alloy surface compared to the CFRP. For a f_z_ = 0.12 mm/blade feed rate, the opposite tendency was observed—lower values of the considered 3D surface roughness parameters were recorded on the CFRP surface. The values of the Sku parameter are less than 3, which means that the surfaces have relative few high peaks and deep valleys. The highest value of the Sds parameter, as in the case of the aluminium alloy, was observed after milling with the feed f_z_ = 12 mm /blade.

Analysis of the 3D maps of the aluminium alloy after machining using the CFRP/Al strategy also reveals a targeted and periodic surface deformation (Figure 9). The lowest values of Sp, Sz, and Sv parameters were obtained after milling with a feed rate of f_z_ = 0.04 mm/blade, while the highest values were obtained after machining with a feed rate of f_z_ = 0.08 mm/blade. A feed rate of f_z_ = 0.12 mm/blade caused a decrease in the values of the considered 3D roughness parameters in comparison with the results obtained for a feed rate of f_z_ = 0.08 mm/blade. Only for the f_z_ = 0.08 was the height distribution spiked (Sku > 3). The highest summit density of peaks was obtained after milling with the feed rate of f_z_ = 0.12 mm/blade.

For the surface topography of the CFRP after machining using the CFRP/Al strategy and feed rates f_z_ = 0.08 mm/blade and f_z_ = 0.012 mm/blade, a directed, periodic distribution of micro-roughness can be observed, which is more pronounced for the surface machined at a feed rate of f_z_ = 0.08 mm/blade (Figure 10b,c). For the sample milled with the lowest feed rate, a surface with a random distribution of micro-irregularities was obtained (Figure 10a). While examining the values of the 3D surface roughness parameters, it was observed that an increase in the value of the feed rate was associated with a decrease in the values of the Sp, Sz, and Sv parameters. It was noticed that on the surface of the aluminium alloy, compared to the CFRP, higher values of the 3D surface roughness parameters were obtained only after the milling with the feed rate of f_z_ = 0.08 mm/blade. For the remaining feed values, a higher 3D surface roughness was obtained on the surface of the CFRP composite compared to the aluminium alloy. Only for f_z_ = 0.08 mm/tooth, Sku > 3, which means that the surface has many high peaks and deep valleys. The highest summit density was also obtained for this value of the feed.

**Figure 11:**
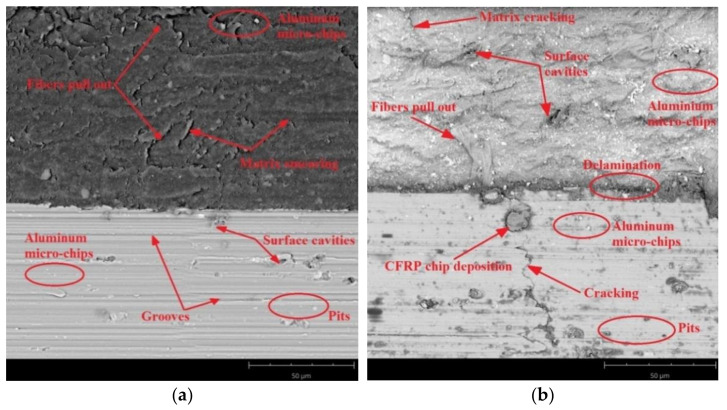
SEM images of aluminium alloy and CFRP surfaces after milling with a feed rate of f_z_ = 0.04 mm/blade and strategy: (**a**) Al/CFRP, (**b**) CFRP/Al.

**Figure 12:**
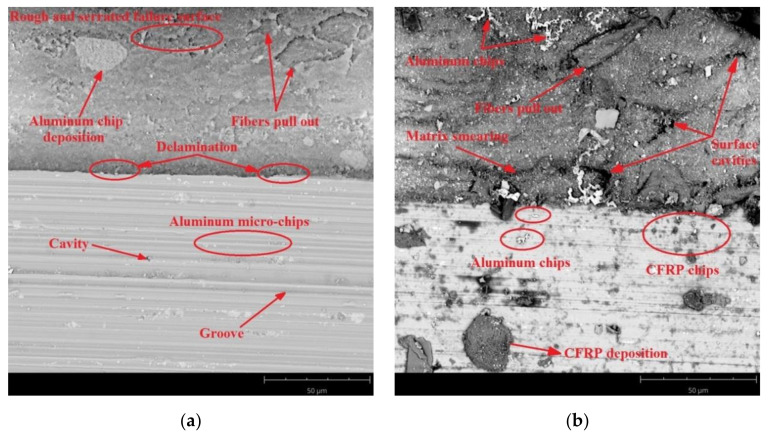
SEM images of aluminium alloy and CFRP surfaces after milling at feed rate f_z_ = 0.08 mm/blade and using the strategy: (**a**) Al/CFRP, (**b**) CFRP/Al.

**Figure 13:**
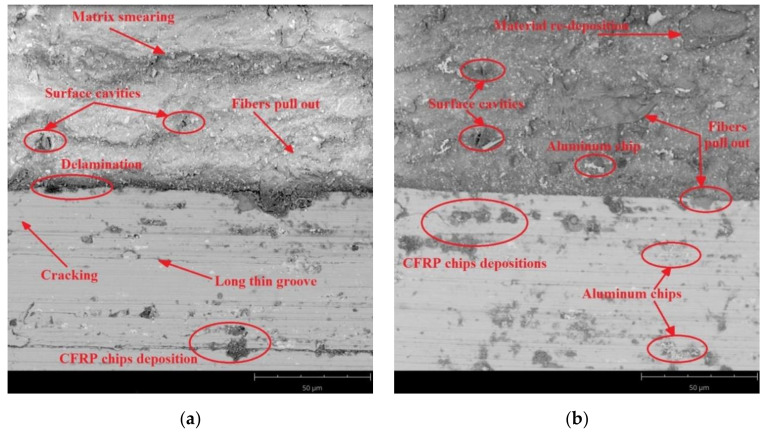
SEM images of aluminium alloy and CFRP surfaces after milling at a feed rate of f_z_ = 0.12 mm/blade and using strategy: (**a**) Al/CFRP, (**b**) CFRP/Al.

SEM images were taken to identify the accuracy of the processing. Figure 11, Figure 12 and Figure 13 present SEM images of the surface obtained depending on the adopted cutting conditions.

Figure 11 shows surface defects of the aluminium alloy and CFRP after milling at a feed rate of f_z_ = 0.04 mm/blade for the two considered machining strategies. The surface of the CFRP after milling using the Al/CFRP strategy shows pulled fibres and cracks in the material caused by it (Figure 11a). The surface of the aluminium alloy shows numerous grooves parallel to the direction of feed as well as surface cavities and pitting (Figure 11a). The composite layer of the sample milled using the CFRP/Al strategy also features pulled fibres. In the analysed case, one can also see the pressed-in micro-chips coming from the metal layer (Figure 11b). The structure has delaminated at the bond between the materials. For the aluminium alloy, one can observe transverse cracking of the material and a significant proportion of embedded chips coming from the composite layer (Figure 11b).

The surface of the CFRP after milling at a feed rate of f_z_ = 0.08 mm/blade and using the Al/CFRP strategy shows warp cracking due to pulled fibres. In addition, the surface shows some pressed-in aluminium micro-chips and particles (Figure 12a). At the joints between the layers, cavities and delamination of the structure are visible, which may have been due to the use of an overly stiff adhesive. The surface of the aluminium alloy shows longitudinal grooves and pressed-in aluminium micro-chips (Figure 12a). For the sample processed using the CFRP/Al strategy, numerous defects can be observed on the surface of the CFRP, including pulled carbon fibres, cavities in the material, smearing of the warp, and embedded aluminium chips. The surface of the metal layer is characterised primarily by the presence of large composite particles embedded in the metal layer (Figure 12b).

Figure 13 presents SEM images of the aluminium alloy and CFRP surfaces after milling with a feed rate of f_z_ = 0.12 mm/blade. The predominant defects occurring on the CFRP surface of a sample machined using the Al/CFRP strategy are pulled fibres, delamination, and smearing of the warp. These defects are most visible at the borders of the layers. The surface in question also shows the presence of aluminium microfibres (Figure 13a). The main defects on the surface of the aluminium alloy are pressed-in composite material, cavities, and pitting (Figure 13a). Similar results were obtained for the CFRP/Al strategy. In addition to pulled fibres and cavities, the surface of the CFRP shows embedded particles of material removed during the machining. On the aluminium surface, similarly to the sample milled using the Al/CFRP strategy, there are numerous insertions in the form of aluminium and composite chips (Figure 13b).

## 4. Discussion

The study evaluates the effect of feed rate and milling strategy on surface roughness and topography after machining a metal–polymer composite sandwich structure using a diamond tool (PCD). The presented results (Figure 5) indicate the occurrence of a different geometrical structure of the surface of the aluminium alloy and the CFRP after treatment. The difference in surface roughness in the examined case results from the heterogeneity of the sandwich structure—during milling, the properties of the materials that make up the structure were altered, which generated variable cutting resistances and different material removal mechanisms. The metal layer that lies above the composite layer caused pressing of the machined object; therefore, the start of milling in the Al/CFRP strategy could lead to a lower surface roughness of the object compared to the CFRP/Al strategy. Furthermore, this could also be due to the stiffening effect of the aluminium covering—the metal layer stiffened the tool, which made the machining process more stable. In addition, the different tensile and compressive stiffness of the sandwich materials resulted in different elastic deformations during cutting. This generated varying surface roughness for the two materials [22].

Higher values of Ra and Rz parameters were obtained on the surface of the CFRP compared to the aluminium alloy. This is due to the poorer machinability of the composite layer resulting from the anisotropy and abrasive nature of the composite material [17]. The higher surface roughness of the CFRP can also be explained by the occurrence of typical forms of damage to this type of material during processing, such as pulling and undercutting of fibres, or delamination [23].

The minimum values of Ra (0.64 µm) and Rz (2.59 µm) surface roughness parameters were obtained on the aluminium alloy surface after milling using a feed rate of f_z_ = 0.04 mm/blade and the CFRP/Al strategy. The highest Ra value (1.67 µm) was observed on the surface of the CFRP after machining with a feed rate of f_z_ = 0.08 mm/blade and using the Al/CFRP strategy. The maximum value of the Rz parameter (6.81 mm/blade) was also obtained on the surface of the CFRP after using a feed rate of f_z_ = 0.12 mm/blade and the CFRP/Al strategy. Maximum values of the analysed surface roughness parameters for different feed rates may be due to the nature of the Ra parameter, which reacts poorly to the local changes that are particularly typical of composite materials.

Studies [24,25,26,27] show that an increase in surface roughness can be associated with an increase in feed rate values. An analysis of the effect of feed rate on the values of Ra and Rz parameters shows that for CFRP machined using the CFRP/Al milling strategy, an increase in feed rate resulted in an increase in the value of the Rz surface roughness parameter. In other cases, the minimum values for both considered parameters were obtained after milling with a feed rate of f_z_ = 0.04 mm/blade. The initial increase in feed rate (f_z_ = 0.08 mm/blade) resulted in an increase in the surface roughness (maximum values of the considered parameters). A further increase in the feed rate (f_z_ = 0.12 mm/blade) led to a slight decrease in surface roughness [28]. The values of Ra parameters on the aluminium alloy surface obtained for all of the considered feed rates were lower after milling using the CFRP/Al strategy compared to the Al/CFRP strategy. A similar trend was observed for the composite layer—the exception being the Ra value obtained when milling with a feed rate of f_z_ = 0.04 blades/mm, which increased in the CFRP/Al strategy. Measurement results for the Rz parameter show that the CFRP/Al strategy resulted in a decrease in the value of this parameter only for the aluminium alloy for f_z_ = 0.04 mm/blade and f_z_ = 0.12 mm/blade. For the composite layer, a lower surface roughness value (for all f_z_ values considered) was obtained using the Al/CFRP strategy [29]. These differences were caused by the different properties of the materials forming the sandwich. The metal layer above the composite layer caused pressing of the machined object. This could also be due to the stiffening effect of the aluminium covering.

The conducted statistical analysis led to the conclusion that only the feed rate had a statistically significant effect on the values of Ra and Rz parameters obtained on the aluminium alloy surfaces. For the CFRP, the values of Ra and Rz parameters were influenced by the feed rate and, to a lesser extent, by the S × f_z_ interaction. Similar conclusions have been reported in [30]. The milling strategy did not statistically significantly affect the surface roughness results in any of the analysed cases.

Analysis of surface topography shows that for most samples, the used tool and the machining conditions produced a directed, periodic pattern of micro-irregularities. The exceptions included the surface topography of the CFRP obtained after using the Al/CFRP strategy and feed rate of f_z_ = 0.04 mm/blade and the surface topography of the CFRP obtained with the CFRP/Al strategy for f_z_ = 0.04 mm/blade. In both cases, in contrast to the 3D maps of the aluminium alloy, surfaces with a random pattern of micro-irregularities and similar heights of vertices and pits were obtained. Such a diverse geometric structure of the surface can affect the durability and intensity of wear during operation. Furthermore, the various surface topographies of the materials making up the sandwich structure can affect the strength of the joints (e.g., adhesive bonds) or the durability and aesthetics of the applied coatings. Analysis of the 3D maps of the milled specimens reveals that the traces of micro-irregularities do not occur at intervals equal to the feed rate (Figure 7, Figure 8, Figure 9 and Figure 10). The most probable reason for this distribution of micro-irregularities was the occurrence of tool radial run-out, which could be caused by the geometry of the cutting tool—when machining with a cutter with a straight toothing, the cross-section of the cut layer changes from 0 to the maximum value, which causes a greater irregularity of load and generation of vibrations.

SEM analysis allowed comparing the surface quality of the tested samples in terms of surface damage. The obtained SEM images indicate that for both machining strategies, increasing the feed rate led to an increase in the proportion of surface defects—the deposition of more micro-particles and chips on the machined surface is noticeable, especially for the CFRP/Al strategy. This may have been due to the axial direction of chip flow resulting from the tool geometry (straight cutter toothing). The observed surface cavities in the composite layer were the result of a local lack of fibres or warp. The visible pitting was the result of the removed material hitting the machined surface. The defects visible on the aluminium alloy surfaces were mainly caused by material pulls and local grain deformation along the machining direction.

The results indicate that the minimum values of Ra, Rz, Sp, Sz, and Sv parameters were obtained on the surface of the aluminium alloy after milling using the CFRP/Al strategy and f_z_ = 0.04 mm/blade. These results could suggest that a low feed rate is most optimal for cutting this type of material with a diamond tool. However, on the other hand, it significantly reduces the efficiency of processing. In the future, it would be advisable to investigate further and check whether the use of a feed rate higher than f_z_ = 0.12 mm/blade would be associated with an increased surface roughness and how the possible increase in surface roughness would proceed.

## 5. Conclusions

The obtained results led to the following conclusions:

1. The feed rate and milling strategy influenced the achievement of different values of the considered surface roughness parameters. However, statistical analysis showed that the surface roughness was statistically significantly influenced by the feed rate and the interaction between feed rate and milling strategy (S × f_z_).
2. Different values of Ra and Rz parameters were observed on the aluminium alloy and CFRP surfaces. Higher values of the discussed surface roughness parameters occurred on the surface of the CFRP.
3. The minimum value of Ra parameter (0.64 µm) was obtained on the aluminium alloy surface of the sample milled at a feed rate of f_z_ = 0.04 mm/blade using the CFRP/Al strategy. The maximum value (1.67 µm) of this parameter was recorded on the CFRP surface after milling at a feed rate of f_z_ = 0.08 mm/blade using the Al/CFRP strategy.
4. The minimum value of Rz parameter (2.59 µm) was obtained on the aluminium alloy surface after machining with a feed rate of f_z_ = 0.04 mm/blade and the CFRP/Al milling strategy. The maximum value (6.81 µm) of this parameter was measured on the surface of the CFRP after using feed rate f_z_ = 0.12 mm/blade and the CFRP/Al strategy.
5. The presented 3D images of the surface show that most of the analysed surfaces were displaying a targeted, periodic distribution of micro-irregularities. Two specimens were the exception, for which the surface topographies of the CFRP (milling with a feed rate of f_z_ = 0.04 mm/blade using the Al/CFRP strategy and milling with a feed rate of f_z_ = 0.04 mm/blade using the CFRP/Al strategy) showed a random distribution of micro-irregularities.
6. The minimum value of Sp parameter (4.67 µm) was obtained on the surface of the aluminium alloy after milling with a feed rate of f_z_ = 0.04 mm/blade and the CFRP/Al strategy. The maximum value of this parameter (11.12 µm) was observed on the surface of the metal layer after machining with a feed rate of f_z_ = 0.12 mm/blade using the Al/CFRP strategy.
7. The minimum values of Sz (7.62 µm) and Sv (2.95 µm) parameters were measured on the surface of the aluminium alloy milled using a feed rate of f_z_ = 0.04 mm/blade and the CFRP/Al strategy. The maximum values for Sz (66.20 µm) and Sv (57.60 µm) parameters were obtained on the surface of CFRP after machining using a feed rate of f_z_ = 0.04 mm/blade and the CFRP/Al strategy.
8. An analysis of SEM images has revealed the presence of numerous defects on the surfaces of layers of the examined samples. Increasing the feed rate led to more chips being deposited on the machined surfaces during machining. For half of the analysed samples, delamination was observed at the border of the bonded materials: (CFRP/Al and f_z_ = 0.04 mm/blade; Al/CFRP and f_z_ = 0.08 mm/blade; Al/CFRP and f_z_ = 0.12 mm/blade).

## Figures and Tables

**Table 1:** ANOVA variance analysis results for the Ra surface roughness parameter.

Effect	Al				
	Sum of Squares	Df	Mean Square	F-Ratio	*p*-Value
S: milling strategy	0.01	1	0.01	0.12	0.73
f_z_: feed	1.15	2	0.58	10.26	<0.01
S× f_z_ interaction	0.03	2	0.01	0.23	0.80
Error	0.67	12	0.06		
Total	1.86	17			
Effect	CFRP				
	Sum of Squares	Df	Mean Square	F-Ratio	*p*-Value[M1] [E.D2]
S: milling strategy	<0.01	1	<0.01	0.41	0.53
f_z_: feed	0.43	2	0.22	27.95	<0.01
S× f_z_ interaction	0.08	2	0.04	4.98	0.03
Error	0.09	12	0.01		
Total	0.60	17			

**Table 2:** ANOVA variance analysis results for the Rz surface roughness parameter.

Effect	Al				
	Sum of Squares	Df	Mean Square	F-Ratio	*p*-Value
S: milling strategy	0.46	1	0.46	0.58	0.46
f_z_: feed	20.15	2	10.07	12.70	<0.01
S× f_z_ interaction	0.26	2	0.13	0.17	0.85
Error	9.52	12	0.79		
Total	30.39	17			
Effect	CFRP				
	Sum of Squares	Df	Mean Square	F-Ratio	*p*-Value
S: milling strategy	0.50	1	0.50	3.50	0.09
f_z_: feed	7.83	2	7.83	27.22	<0.01
S× f_z_ interaction	1.29	2	0.64	4.48	0.04
Error	1.73	12	0.14		
Total	11.35	17			

## Data Availability

The raw/processed data required to reproduce these findings cannot be shared at this time due to technical or time limitations. Data can be made available on individual request.
